# Comparing CT perfusion with oxygen partial pressure in a rabbit VX2 soft-tissue tumor model

**DOI:** 10.1093/jrr/rrt092

**Published:** 2013-09-26

**Authors:** Chang-Jin Sun, Chao Li, Hai-Bo Lv, Cong Zhao, Jin-Ming Yu, Guang-Hui Wang, Yun-Xiu Luo, Yan Li, Mingyong Xiao, Jun Yin, Jin-Yi Lang

**Affiliations:** 1 Department of Radiation Oncology, Chengdu Third People's Hospital, Sichuan, 610041, China; 2 Department of Head and Neck Surgery Oncology, Sichuan Cancer Hospital, Sichuan, 610041, China; 3 Department of Radiation Oncology, Shandong Cancer Hospital, No. 8 Zhenyunling Road, Chengdu, Sichuan, 610202, China; 4 Department of Radiation Oncology, Sichuan Cancer Hospital, Sichuan, 610041, China; 5 Department of Radiology, Civil Aviation Medical Center, Chengdu, Sichuan, 610041, China

**Keywords:** perfusion computed tomography, rabbit model, VX2 tumor, oxygen partial pressure, hypoxia

## Abstract

The aim of this study was to evaluate the oxygen partial pressure of the rabbit model of the VX2 tumor using a 64-slice perfusion CT and to compare the results with that obtained using the oxygen microelectrode method. Perfusion CT was performed for 45 successfully constructed rabbit models of a VX2 brain tumor. The perfusion values of the brain tumor region of interest, the blood volume (BV), the time to peak (TTP) and the peak enhancement intensity (PEI) were measured. The results were compared with the partial pressure of oxygen (PO2) of that region of interest obtained using the oxygen microelectrode method. The perfusion values of the brain tumor region of interest in 45 successfully constructed rabbit models of a VX2 brain tumor ranged from 1.3–127.0 (average, 21.1 ± 26.7 ml/min/ml); BV ranged from 1.2–53.5 ml/100g (average, 22.2 ± 13.7 ml/100g); PEI ranged from 8.7–124.6 HU (average, 43.5 ± 28.7 HU); and TTP ranged from 8.2–62.3 s (average, 38.8 ± 14.8 s). The PO2 in the corresponding region ranged from 0.14–47 mmHg (average, 16 ± 14.8 mmHg). The perfusion CT positively correlated with the tumor PO2, which can be used for evaluating the tumor hypoxia in clinical practice.

## INTRODUCTION

Hypoxic cells, which are present in almost all solid tumors, are not sensitive to radiotherapy and are an important reason why these tumors are difficult to cure, reoccur easily, and have poor prognosis [1–4]. Studies in the literature have shown that tumors must have abundant blood vessels, a rich blood supply, and a high oxygen content to have high radiosensitivity so that the therapeutic effect will be good; otherwise, the therapeutic effect will be poor. Accurate assessment of tumor blood supply can help assess the tumor oxygen supply [5–7]. However, tumor blood supply and oxygen supply are difficult to measure in clinical practice, and the tumor vasculature observation (intercapillary distance and microvessel density [MVD]) cannot reflect the whole picture of the tumor, and sampling error cannot be avoided. Also, the method is cumbersome and time-consuming. Measurement with an oxygen electrode can directly reflect the tumor's oxygen-containing status and this method is considered to be the ‘gold standard’ for evaluating the tumor hypoxia status. However, it is an invasive diagnostic method and it can only be applied to superficial tumors [1–3]. A variety of noninvasive methods including single photon emission computed tomography (SPECT), fluoromisonidazole (FMISO) positron emission tomography (PET), and electron paramagnetic resonance imaging have been tried for the assessment of tumor hypoxia; however, all of these methods have shortcomings [1–4, 8, 9]. XeCT and PET have also been used to evaluate the tissue blood supply. However, the spatial resolution is low, and there are limitations with regard to displaying the tissue structure. Also, clinical reproducibility is low [5, 9].

Among the traditional imaging tools, MR can provide finer anatomical images than CT. However, in the modern 3D treatment planning system (3DTPS), CT images reflect the density changes of the tissues, and the accuracy of the dose calculation and distribution is closely related to the CT values of the tissue structure that are input into the system. The CT images are the basic images for planning, and perfusion CT images can be directly merged with conventional CT scan images. There is no problem with the registration accuracy. In addition, MR perfusion imaging can only provide the relative values of perfusion parameters, but perfusion CT can provide the absolute values of perfusion parameters. Therefore, CT is more suitable for this study than MR [10].

Perfusion CT is an imaging examination technique that obtains the characteristic physiological parameters of tumor blood supply or blood supply of other tissues using the current CT technique and the traditional contrast enhancement method. It can provide a series of parameters that can be calculated and analyzed, such as blood volume (BV) and blood flow (BF), that can be used for the comprehensive evaluation of perfusion status and tissue vascular level in the tumor tissue, reflecting microscopic physiological changes consistent with tumor angiogenesis [5–7]. A large number of studies have shown that tumor partial pressure of oxygen (PO2) is closely related to its blood flow [10–12]. There are limits to the use of perfusion CT in studying the relationship between human tumor PO2 and perfusion parameters directly, and it is difficult to achieve point-to-point acquisition of the PO2 value and perfusion CT parameters. Perfusion CT parameters have been measured successfully in rabbit VX2 soft tissue tumors [13, 14]. VX2 tumors of rabbits are similar to human head and neck squamous cell carcinoma, liver cancer, and other solid tumors with invasion and lymph node metastasis, and angiogenesis, and have other molecular biological characteristics that are similar. In a previous study, we showed that perfusion CT could be used for evaluating the target volume for 3D conformal radiotherapy in the rabbit VX2 brain tumor model [15]. In this study, we used the rabbit model of a VX2 soft-tissue tumor to explore the relationship between the functional parameters of perfusion CT and the PO2 of the corresponding region of interest and compared the results with those obtained using the oxygen electrode method. Our study may provide a basis for the further use of this non-invasive method of evaluating tumor PO2 status.

## MATERIALS AND METHODS

### Experimental animals

Pure-bred New Zealand white rabbits of either sex weighing 2–3 kg were provided by the Animal Center of Sichuan Academy of Medical Sciences (license number SCXK (Shanghai) 2003-0003 Shanghai Slack Experimental Animal Co., Ltd). The VX2 tumor cells were obtained via intramuscular injection of the hind legs of breeding rabbits with a tumor *in vivo*. The breeding rabbits were provided by the Institute of Ultrasound, Chongqing Medical University.

### Model construction

#### Anesthesia

The animals were anesthetized by intramuscular injection of 0.3–0.4 ml/kg Sumianxin II.

#### Preparation of VX2 cell suspension

The solid tumors in the groin area of rabbits injected with VX2 tumor cells were peeled off under anesthesia and aseptic surgical conditions and then rinsed with normal saline. The envelope was removed, and the fish flesh-like tissue with vigorous growth at the tumor edge was collected and cut into approximately 2-mm^3^ pieces. The samples were washed by centrifugation in Hank's solution at 1000 rpm for 5–10 min, and the tissue blocks were placed in a 200–300 mesh cell sieve homogenizer and homogenized with an appropriate amount of 0.9% normal saline containing gentamicin. The cell suspension was collected and washed by centrifugation at 1000 r/min for 5 min. Serum-free RPMI-1640 solution was used to dilute the cell concentration to 1 × 10^7^/ml, and a cell suspension was prepared. A trypan blue exclusion assay was used to determine that the number of viable cells was >95%, and cells were made into a 10^7^/ml cell suspension.

#### Tumor cell implantation

The experimental rabbits were fixed in the prone position after anesthesia. The hind-leg hair was routinely cut and the skin was disinfected. A 1-ml syringe with a No. 6 needle was used to aspirate a 0.5-ml prepared cell suspension, and the needle was inserted into the vastus lateralis of the rabbit's right thigh. The needle depth was 0.5 cm, and the injection site was pressed for about 1 min after needle withdrawal to prevent bleeding and tumor cell overflow. Conventional 400 000–600 000 units of penicillin were administered after surgery, and rabbits underwent intramuscular injection of gentamicin (40 000 units for three days).

#### Signs of tumor formation

The marker of tumor survival was detection of a right thigh nodule 10–18 days after implantation. If no nodule was found, then no tumor formation was defined.

### General information

One of the 46 New Zealand rabbits did not undergo scanning successfully 12–14 days after implantation due to poor anesthetic effect and movement during scanning; 45 animals underwent scanning successfully and were included in the final data analysis.

### Instruments and methods

#### Measurement of PO2

The OxyLab pO_2_^TM^ oxygen partial pressure instrument (PowerLab 2/25 AD Instruments, Model: ML 825 Serial: 225-0508 manufactured by AD Instruments, Pty Ltd and provided by Shandong Cancer Hospital) was used. The PO2 instrument was turned on and the oxygen fiber probe was connected. The CT bed position with the maximum tumor area visible was recorded; the same tip system was used to move the bed to that level, and the detector laser light was turned on. In order to facilitate the insertion of the oxygen fiber probe, a 21G hollow needle was inserted first along the CT laser positioning line to guide the insertion of the oxygen probe. Readings would be unstable when the probe was just inserted, therefore, we usually waited for 1–5 min and recorded the reading after it had been stabilized for 10 s. That reading was the PO2 of that point. The partial pressure values and the corresponding perfusion parameters of 45 points in 45 experimental rabbits were measured.

#### CT images measurements

A Philips Brilliance 64-slice spiral CT scanner (Philips Medical Systems, Cleveland, OH) was used. A Medrad-Stellant CT high-pressure auto-injector was used to inject non-ionic contrast agent (iohexol, 300 mg/ml, GE pharmaceutical company) via the lateral thigh vein at a flow rate of 5 ml/s; the injection volume was 1.5–2 ml/kg. Saline was injected immediately after contrast agent injection.

Conventional cross-sectional CT scanning was performed first with the following scan parameters: tube voltage 80 kV, current 120 mA, matrix 512 × 512, field of vision (FOV) 250 mm, slice thickness 2.5 mm.

In the axial unenhanced CT images, the slice with the maximum tumor area visible was selected as the center slice for perfusion CT. The perfusion CT scan parameters were as follows: after the PO2 detected by the PO2 instrument was recorded the oxygen probe was withdrawn, but the 21G hollow needle was retained in the same place. CT scanning was again carried out and the position, tilt angle and depth of the indwelling of the hollow needle were measured on the CT images. The location of the hollow needle in the functional image of the corresponding layer was found and the ROI was placed at that position. The ROI size was similar to that of the probe. The femoral artery was selected as the input artery. Values for BF, BV, TTP and PEI were recorded.

The perfusion CT scan procedure was selected, and in-layer dynamic scanning was performed with the following parameters: the scanning mode was sequence, the slice thickness was 2.5 mm, 120 kV, 84 mA, collimator 0.625 mm, scan time 1 s/360°. Image acquisition was once every 2 s, and the scan was continued for 60 s. The delay time was 0 s. A total of 16 sequences were obtained; each sequence contained 30 images.

#### Image processing

The obtained images were transferred to a Philips EBW CT image-processing workstation (Philips Healthcare; DA Best, Netherlands) and a commercial perfusion software package (Brilliance perfusion 2.1.1) was used [10, 16]. The maximum-slope (MS) method was applied for the algorithms of Perfusion CT. The MS method of deriving perfusion measurement was proposed by Miles *et al.* [16, 17]. In this method there is an input artery (the femoral artery was selected as the input artery) but no venous outflow. In this circumstance, the target tissue CT value increases proportionally as the transferred contrast medium volume increases. Tissue blood flow was calculated as the maximum slope of the tissue time attenuation curve divided by the maximum height of the arterial time attenuation curve [16–18]. Four kinds of images were generated including the corresponding layer perfusion, blood volume (BV), time to peak (TTP), and peak enhancement intensity (PEI).

#### Statistical analysis

All data are shown as mean ± SD. Pearson correlation analysis was used to study the relationship between perfusion CT parameters and PO2. The definition of hypoxia was PO2 < 5 mm, and the potential cutoff values were tested. ROC curve analysis was used to determine the cutoff value [4, 19]. Data were analyzed using SPSS 15.0 statistics software (SPSS Inc., Chicago, IL), and a *P*-value < 0.05 was considered statistically significant.

## RESULTS

### Relationship between the CT perfusion parameters of rabbit VX2 tumors and PO2

**Fig. 1:**
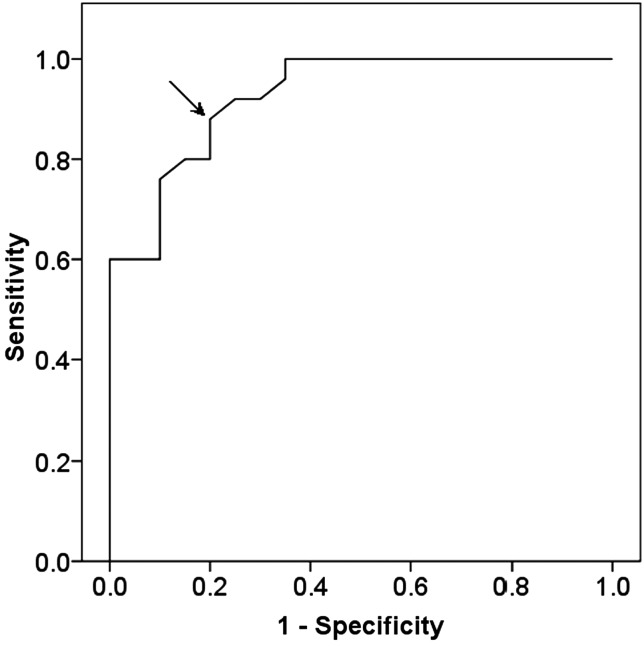
The perfusion value of 8.9 ml/min/ml (arrow) was selected as the cutoff point for the diagnosis of VX2 tumor hypoxia.

According to the literature, PO2 < 5 mmHg is defined as hypoxia. It can be seen from Fig. 1 that the cutoff point of the BF value for the diagnosis of hypoxia of VX2 tumor was 8.9 ml/min/ml, which optimized both a sensitivity of 0.88 and specificity of 0.80.

**Fig. 2.**
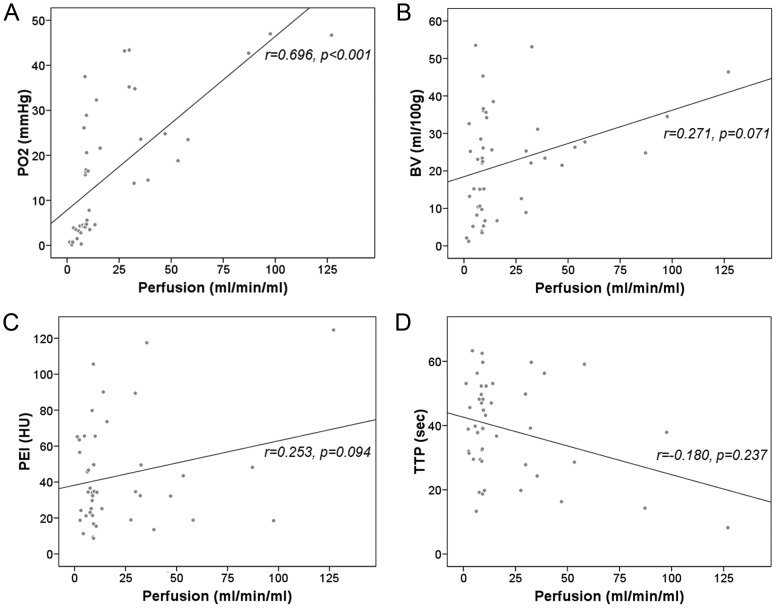
Relationships between perfusion CT parameters of rabbit VX2 tumors and (**A**) oxygen partial pressure (PO2), (**B**) blood volume (BV), (**C**) peak enhancement intensity (PEI), and (**D**) time to peak (TTP).

Table 1 and Fig. 2 show that the correlation coefficient between the BF values of the rabbit VX2 tumors and the PO2 of the corresponding region was 0.696 (*P* < 0.0001, Fig. 2A). The correlation coefficients between the PO2 of the corresponding region and BV, PEI and TTP were 0.271, 0.253 and − 0.18, respectively, which were not statistically significant (*P* = 0.071, *P* = 0.094 and *P* = 0.237, respectively, Fig. 2B, C and D).

**Table 1.** CT perfusion parameters of rabbit VX2 tumors and oxygen partial pressure

	Range	Mean ± SD	Pearson's correlation	*P*
PO2 (mmHg)	0.14–47	16 ± 14.8		
Perfusion (ml/min/ml)	1.3–127	21.1 ± 26.7	0.696	<0.0001
BV (ml/100g)	1.2–53.5	22.2 ± 13.7	0.271	0.0714
PEI (HU)	8.7–124.6	43.5 ± 28.7	0.253	0.0938
TTP (sec)	8.2–63.3	38.8 ± 14.8	−0.180	0.2373

### Determination of the cutoff point of the perfusion value for the diagnosis of hypoxia for a VX2 tumor (PO2 < 5 mmHg)

#### Diagnostic value of the perfusion values for hypoxia of a VX2 tumor (PO2 <5mmHg)

**Fig. 3.**
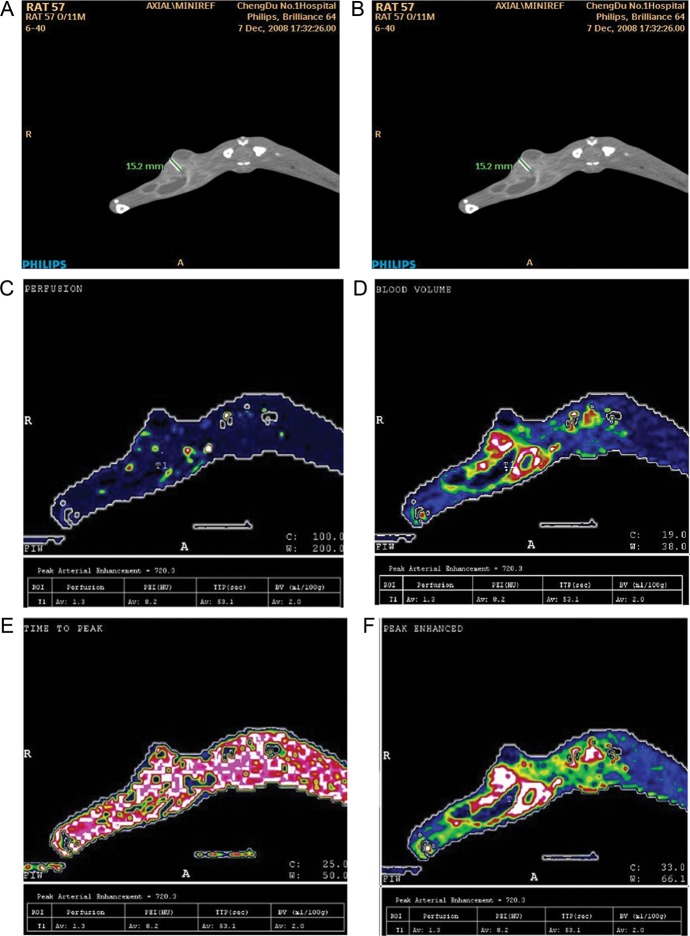
Measurement of PO2 and acquisition of functional parameters of the same region of interest in one animal; PO2 < 5 mmHg. (**A**) PO2 at region of interest was 0.75 mmHg as measured by the oxygen electrode; (**B**) based on Figure 1, corresponding regions of interest are selected in the same layer of the original image; (**C**) perfusion map perfusion = 1.3 ml/min/ml); (**D**) blood volume map BV = 2.0 ml/100g); (**E**) time to peak map TTP = 53.1 sec) (**F**) peak enhancement intensity map PEI = 8.2 HU.

**Fig. 4.**
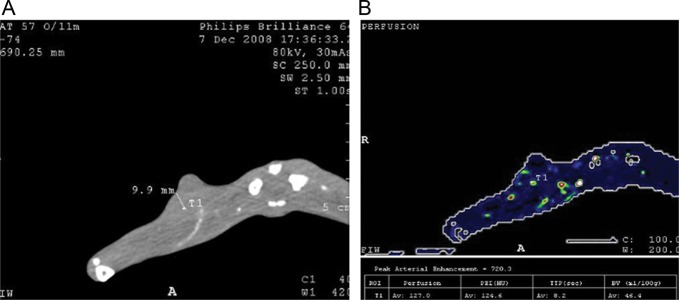
Measurement of PO2 and acquisition of functional parameters of the same region of interest in one animal, PO2 > 5 mmHg. (**A**) The oxygen electrode measured the PO2 at that region of interest (PO2 = 46.7 mmHg). (**B**) Perfusion map (perfusion = 127.0 ml/min/ml).

Table 2, Fig. 3 and Fig. 4 show the diagnostic value of the perfusion value for hypoxia of a VX2 tumor when the perfusion value of 8.9 ml/min/ml was used as the cutoff point. In 25 rabbits, PO2 was < 5 mmHg (Fig. 3 shows the same animal); in 20 rabbits, PO2 was > 5 mmHg (Fig. 4 shows the same animal); in 3 rabbits, PO2 was < 5 mmHg while perfusion values were > 8.9 ml/min /ml; in 4 rabbits, PO2 was > 5 mmHg while perfusion values were < 8.9 ml/min/ml.

**Table 2:** Diagnostic value of the perfusion values for hypoxia of VX2 tumor

Cut point	Sensitivity	Specificity	Positive predictive value	Negative predictive value	Accuracy
Perfusion	0.88	0.80	0.85	0.84	0.84
(8.9 ml/min/ml)	(22/25)	(16/20)	(16/19)	(22/26)	(38/45)

## DISCUSSION

Our study systematically evaluated the relationship between various perfusion CT functional parameters and the PO2 of VX2 tumors and found that perfusion values of rabbit VX2 tumors were significantly positively correlated with the PO2. The BV, PEI, and TTP values were not significantly correlated with perfusion values. Our results suggest that perfusion values reflect the PO2 in the tumor. The faster the tumor blood flow, the more oxygen is brought by blood flow and the smaller the probability of tissue hypoxia.

Our results are consistent with most reports in the literature [5, 6]. One exception is a report by Hemphill *et al*. who used a perfusion CT to study the correlation between the various perfusion parameters of patients with brain trauma and PO2, and found that BF and BV values were not correlated with PO2 (GE deconvolution algorithm software was used, and the BF value is equivalent to the perfusion values in the slope method) [20]. Their study found that the mean transit time (MTT) was negatively correlated with the PO2. Possible reasons for the conflicting results are: (i) differences in study subjects; and (ii) the BF value of the brain gray matter was mistakenly calculated while measuring the BF value of the brain white matter [5]. We used the slope method perfusion software of Philips Medical Systems, which cannot provide the MTT value. Therefore, we did not analyze the correlation between the MTT values of rabbit VX2 tumors and PO2. The slope method perfusion software from Philips has been widely used in cancer research because of the short scan time (even shorter than that for MTT). The drawback of the slope method is the exact ‘cutoff’ point of the slope. If there is a need to obtain a large slope, this requires an increase in the injection volume and injection rate, which may affect the patient's cardiac output. As for the correlation between the BV values and PO2, our study and their study drew the same conclusion, indicating that although the density of local blood vessels is great at some parts of the tumor, tumor hypoxia may occur due to tortuous new blood vessels, poor blood flow, and insufficient oxygen exchange. Although the two kinds of software use different algorithms, a recent study on cervical cancer by Shibuya *et al*. showed that the two algorithms produced the same result for the correlation between blood flow and PO2 [18]. Therefore, the results of Shibuya *et al*. and those of Hemphill *et al*. are comparable.

Our results showed that the correlation coefficient between the BF values of rabbit VX2 tumors and the PO2 of the corresponding region was 0.696, and therefore the correlation coefficient is still not very high. The possible causes are: (i) although in our study we used CT-guided puncture and a guide needle was positioned, and the oxygen electrode was placed via the guide needle, which ensures the accuracy of measurement to the maximum extent, slight displacement during the piercing process of the guide needle is inevitable; and (ii) the internal environment is complex, leading to many factors that may cause tumor hypoxia. The blood-flow velocity is only one of the important factors that may affect PO2.

Mayr *et al*. adopted the relative MR signal intensity (the highest signal strength of a given tissue after enhancement divided by the signal strength before enhancement, RSI) to express tumor perfusion [7]. Their study of 17 cases of cervical cancer showed that areas with hypoxia and poor blood supply are not only located at the tumor center, as is commonly thought, but are distributed throughout the tumor. Different tumor components have different blood supply and oxygen supply, and the average RSI of the tumor central area cannot reflect the overall blood supply and oxygen supply of the tumor. Certain RSI 10% sites are significantly associated with tumor recurrence. If the region with RSI ≤ 2.5 reached 10% of the total tumor volume, the tumor recurrence rate after radiotherapy was 88%. If the region with RSI ≤ 2 reached 10% of the total tumor volume, the tumor recurrence rate after radiotherapy was 100%. Therefore, regions with RSI ≤ 2.5 can be considered the ‘hypoxic area’ of the tumor. Jansen *et al*. used enhanced MR scanning and ^18^F-FMISO PET-CT to compare 18 metastatic lymph nodes in the head and neck in 13 patients and found that hypoxic lymph nodes had lower perfusion values compared with non-hypoxic lymph nodes [21]. The aforementioned clinical studies using MR imaging suggest that accurate measurement of tumor perfusion values is of significance for assessing tumor hypoxia.

In our study, the Phillips 64-slice CT was used, which covered 4 cm of anatomy per rotation. The whole tumor perfusion cannot be carried out, therefore, its use in assessing the tumor hypoxia percentage (HP) is limited, which restricts its application in radiotherapy planning. However, if the jog technique is used, the *z*-axis length can be up to 8 cm. The latest 320-slice CT scanner can cover 16 cm of anatomy in the *z*-axis direction per rotation, which allows it to perform perfusion imaging for tumors of any size. Technical development has made the use of perfusion imaging possible in measuring tumor HP and delineating the hypoxic target for radiotherapy [22].

The limitations of this study were: (i) samples of the tumor tissues from the ROI were not obtained for the test of immunohistochemical markers of hypoxia, therefore, hypoxia at that point was not further proved from the perspective of immunohistochemistry; and (ii) tumor hypoxia is generally assessed using tumor HP, i.e. the percentage of actually measured tumor PO2 data that are <2 mmHg (1 mmHg = 0.1333 kPa), <5 mmHg or <10 mmHg (represented by HP2, HP5 and HP10, respectively) [23]. As we only selected the layer of interest and performed point-to-point analysis, we could not assess the ratio of perfusion values, and compare them with the HP. Therefore, our results only suggest a relationship between the amount of blood flow at that point of interest with the PO2 measured by the oxygen probe, and cannot prove that the perfusion values obtained from perfusion CT are correlated with hypoxia.

## CONCLUSION

In summary, the results of this study showed that perfusion values obtained from perfusion CT are positively correlated with tumor PO2, and that perfusion CT has the potential to be applied in assessing tumor hypoxia in clinical practice. It can provide a basis for delineating the hypoxic target area in 3D conformal radiotherapy.

## FUNDING

Funding for this research was provided by the National Natural Science Foundation of China (NSFC) (No. c30670618), and the Science Technology Funds for Young Scholars of Sichuan Province (No. 09ZQ026-065).
